# Calcium-Dependent RTX Domains in the Development of Protein Hydrogels

**DOI:** 10.3390/gels5010010

**Published:** 2019-02-25

**Authors:** Beyza Bulutoglu, Scott Banta

**Affiliations:** Department of Chemical Engineering, Columbia University, 500 W 120th Street, New York, NY 10027, USA; bbulutoglu@mgh.harvard.edu

**Keywords:** RTX domain, beta roll domain, environmentally responsive hydrogels, calcium-dependent folding, responsive biomaterials

## Abstract

The RTX domains found in some pathogenic proteins encode repetitive peptide sequences that reversibly bind calcium and fold into the unique the β-roll secondary structure. Several of these domains have been studied in isolation, yielding key insights into their structure/function relationships. These domains are increasingly being used in protein engineering applications, where the calcium-induced control over structure can be exploited to gain new functions. Here we review recent advances in the use of RTX domains in the creation of calcium responsive biomaterials.

## 1. Introduction

Hydrogel materials that are responsive to environmental stimuli, such as changes in pH, temperature, and solvent composition, are being explored for use in important applications, including gene and drug delivery, tissue and cell culture, and in the development of biosensors. Several excellent reviews are available on different types of crosslinking moieties that are responsive to environmental cues and their applications [1,2,3,4,5,6]. For example, elastin-like polypeptides can form reversible aggregates above their transition temperatures [7,8], and α-helical coiled-coil leucine zipper domains can form cross-links via hydrophobic interactions and pH-dependent interactions of charged amino acid residues [9,10]. Other protein domains, such as the EF-hand (helix-loop-helix) motif of calmodulin, are responsive to binding of small molecules, such as calcium, which results in a conformational change of the protein domain, allowing for ligand binding and increased crosslinking [11,12]. Additional examples include hydrogels constructed with designed MAX peptides with lysine residues, which form β-hairpins under basic conditions resulting in gelation [13,14], and the FEK16 peptide (16-mer rich in phenylalanines, glutaminc acids, and lysines), which forms β-sheets upon addition of NaCl, CaCl_2_, and KCl [15]. 

The Repeats-in-Toxin (RTX) domains undergo a conformational change and fold into the β-roll secondary structure specifically upon calcium binding. Proteins with RTX domains are produced by a variety of Gram-negative bacteria and these domains are employed in the translocation of the protein across the membrane [16]. As such, RTX domains gained attention in toxicology research and many studies have been published on the mechanism of RTX folding, and on their structure-function relationships [17,18,19,20]. In recent years, the reversible conformational change of these domains have been explored for biotechnology applications, including the development of protein hydrogels where the gelation can be controlled via adjustment of calcium concentrations [21,22,23]. This is a unique biomaterials approach, as the calcium ions do not participate in the cross-linking directly, but instead calcium ions specifically enable the formation of a protein structure (the β-roll domain), which can then be engineered to form cross-linking and other useful biomolecular interfaces. In this review, we will briefly introduce RTX domains and review recent studies where these domains were utilized as responsive biomaterial building blocks.

## 2. Repeats-in-Toxin (RTX) Domains and the β-Roll Fold

Proteins with repeats-in-toxin (RTX) domains represent a large family of virulence factors, with over 1000 family members, produced by Gram-negative bacteria with diverse biological functions. These domains are present in many lipases (e.g., lipase A from *Serratia marcescens*) and proteases (e.g., alkaline protease from *Pseudomonas aeruginosa*). RTX proteins share two common features. First, they have repeats of 9 amino acid (nonapeptide) sequences rich in glycine and aspartic acid residues at their C-termini, which are involved in calcium binding. Additionally, they are secreted across the bacterial envelope via the type I secretion system. Typically, the C-terminal repetitive sequence is composed of several blocks of GGXGXDXUX, whereby U represents an aliphatic amino acid and X can be any residue. RTX domains become biologically activated upon calcium binding, as they serve as molecular chaperons or as the folding nuclei. Inside the cell at low calcium concentration (<1 μM), they likely remain in their disordered state. Upon translocation into the extracellular space, the relatively higher calcium concentrations (>1 mM) enable the RTX domains to fold into functional globular proteins. The interaction of most virulence factors, such as *Escherichia coli* α-hemolysin (HlyA), with their target cells depends on the proper secretion and folding mechanism of RTX domains [16,20,24,25,26,27,28,29,30,31]. The dependence of RTX domain activity on calcium binding was first discovered in HLyA of *E. coli* and adenylate cyclase (CyA) from *Bordetella pertussis* [32,33].

**Figure 1:**
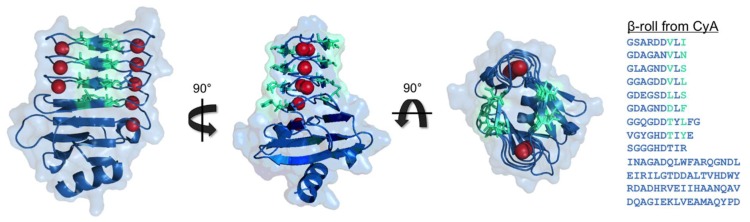
The β-roll from adenylate cyclase (CyA) of *B. pertussis*. The peptide structure is shown in the folded conformation bound to calcium ions (red) (PDB file: 5CVW). The residues forming the β-sheet faces are highlighted in turquoise in the amino acids sequence on the right. The figures were rendered in PyMOL.

Crystal structures of RTX domain-containing proteins led to the discovery that RTX domains bind calcium and fold into a unique β-roll secondary structure motif, which resembles a flattened corkscrew structure. The structure is composed of two parallel β-sheets formed by the XUX amino acids in the RTX consensus sequences. The aliphatic amino acids project inward, forming a hydrophobic core and the X amino acids project outward from the protein sheets. The glycine rich amino acids enable turns between the β-sheets and the conserved aspartic acid residues bind the calcium ions, which are located between the successive turn regions [18,34]. These features can be seen in the Block V RTX domain from the adenylate cyclase (CyA) protein of *B. pertussis*, as shown in Figure 1. It has also been shown that the binding of a calcium molecule assists the conformational change of the adjacent binding site, resulting in cooperativity between binding events and an overall polarized folding mechanism of the domain [18,34]. 

### 2.1. Adenylate Cyclase from Bordetella Pertussis

The CyA protein of *B. pertussis* contains five blocks of repetitive RTX domains. The fifth RTX domain (Block V) has been isolated from the CyA parent protein and has been extensively investigated as a model RTX domain. It is composed of 152 amino acid residues with a molecular weight of 15.9 kDa. This peptide has nine repeats of the nine-residue consensus motif along with a capping group at the C-terminus, which is necessary for proper folding of the isolated peptide [17,18,35,36,37]. The isolated peptide is disordered at low calcium concentrations and folds into the β-roll secondary structure upon calcium addition, as shown in Figure 1. In its folded conformation, the peptide has two β-sheet faces with 8 amino acid side chains that project out radially on each side of the domain, and these amino acids have been extensively mutated without affecting the folding of the β-roll structure. 

### 2.2. Serralysin from Serratia Marcescens and Other RTX Proteins

The serralysin protein of *S. marcescens* is a 50 kDa metalloprotease with an N-terminal catalytic domain with zinc-binding motif HEXXHXXGXXH, where histidine residues are the zing ligands, and a single C-terminal RTX domain composed of 6 repeats of 9 amino acid sequences (nonarepeats) [38,39,40]. The folded RTX domain was shown to initiate the folding and activation of the protease domain. In addition, the interactions across the protease and RTX domains were shown to contribute to the stability of the catalytic N-terminal helix, promoting pathogenic activity [41]. Other than *B. pertussis* and *S. marcescens,* cytotoxic proteins with RTX domains are present in many bacteria including *E. coli*. Among the best-characterized RTX proteins is the alkaline protease from *Pseudomonas aeruginosa*, which is another zinc-dependent metalloprotease with a calcium-dependent RTX domain composed of 6 nonarepeats [27,42,43]; the HlyA protein from *E. coli* has a C-terminal RTX domain with 11 nonarepeats [44].

## 3. RTX Domain-Based Calcium Responsive Hydrogels

RTX domains have been explored as building blocks for creating hydrogel materials, as the calcium dependent disorder-to-order transition can be used as a means of controlling the network assembly and hydrogel formation. Stimulus responsive hydrogels were constructed, whereby RTX domains were utilized in combination with other cross-linking domains, such as leucine zippers and as stand-alone cross-linking domains. 

### 3.1. β-Roll Mutants Fused to α-Helical Leucine Zipper Domain

**Figure 2:**
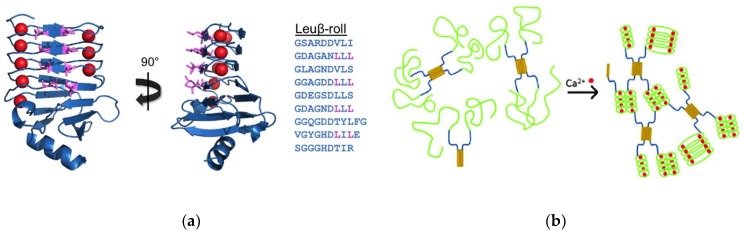
The Block V RTX domain as a hydrogel building block. (**a**) The positions on one β-sheet face have been mutated to hydrophobic leucine residues. The folded conformation of the Leuβ-roll mutant is shown (PDB file: 5CVW) along with the peptide sequence of the β-sheet forming residues, whereby the mutated positions are shown in purple. (**b**) The Leuβ-roll (green) has been fused to an α-helical leucine zipper domain (yellow) separated by a soluble linker (blue). The β-rolls fold upon calcium binding resulting in additional cross-linking leading to network formation. Adapted with permission from Biomacromolecules, 2012, 13(6), pp 1758–1764. Copyright (2012) American Chemical Society.

**Figure 3:**
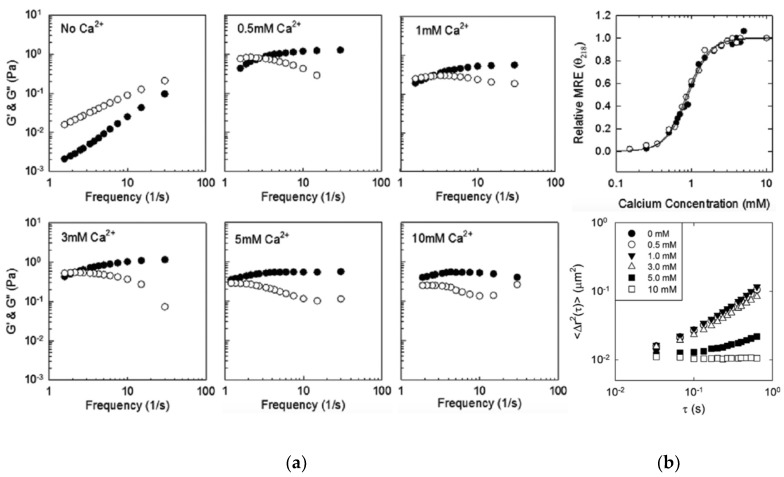
Calcium dependent tuning of the rheological properties of RTX-domain hydrogels. (**a**) Microrheology data of H-S-Leuβ-roll calcium titration with viscous (o) and elastic (●) moduli, showing the transition from viscous sample to elastic hydrogel with increasing calcium concentrations; (**b**) Top panel: circular dichroism data of the WTβ-roll (●) and the Leuβ-roll (o) indicating complete folding at calcium concentrations larger than 3 mM. Bottom panel: Time averaged mean square displacement (MSD) measurements of H-S-DLeuβ-roll calcium titration. A linear increase in MSD with respect to lag time was observed at low calcium concentrations, whereas a plateaued MSD profile indicated hydrogel formation. Adapted with permission from Biomacromolecules, 2012, 13(6), pp 1758–1764. Copyright (2012) American Chemical Society and Biomacromolecules, 2014, 15(10), pp 3617–3624. Copyright (2014) American Chemical Society.

In the calcium-bound, folded conformation, the Block V RTX domain of CyA forms a β-roll domain with a C-terminal capping group with two parallel β-sheet faces, each composed of 4 β-strands with 8 amino acids projecting outward into the solvent (exhibited in turquoise in Figure 1). A hydrophobic interface was created on one side of the β-roll domain by mutating these positions on one of the β-sheet faces to leucine residues (Figure 2a) [45]. This mutant, termed Leuβ-roll, was genetically fused to an α-helical leucine zipper domain (H) separated via a soluble linker domain (S). In the absence of calcium, leucine zipper domains form tetrameric bundles, while the Leuβ-roll domains remain disordered. Upon addition of calcium, the Leuβ-roll domains fold and form leucine-rich hydrophobic interfaces, enabling non-covalent cross-linking via hydrophobic driving forces. This structural transition results in cross-links on both ends of the H-S-Leuβ-roll construct, leading to hydrogel network formation, as demonstrated in Figure 2b. Wild-type (WT) β-roll domains were fused to H and S domains as control polypeptides. Microrheology experiments were performed with H-S-WTβ-roll and H-S-Leuβ-roll in the presence of calcium or magnesium (which does not trigger β-roll formation). Hydrogel formation was only observed with Leuβ-roll in the presence of calcium at concentrations greater than 3–5 mM [45]. It was also shown that the rheological properties of the H-S-Leuβ-roll hydrogel can be tuned with calcium following the calcium dependent folding of the Leuβ-rolls. Upon calcium titration, a transition from a viscous liquid to viscoelastic hydrogels was observed between 0.5 and 5 mM calcium, as indicated by the change in viscous and elastic moduli of the samples (Figure 3a). This transition was in good agreement with the circular dichroism data, which indicated that the β-rolls are completely folded at concentrations greater than 3 mM (Figure 3b). Thus, it was demonstrated that environmentally-responsive hydrogel networks with tunable rheological properties can be constructed using mutant RTX domain capable of cross-linking only in calcium-rich environments.

**Figure 4:**
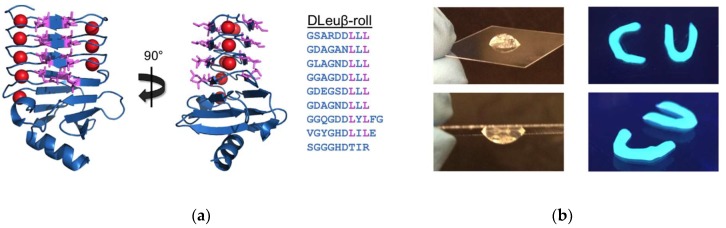
The DLeuβ-roll as a stand-alone hydrogel building block. (**a**) The positions on both β-sheet faces were mutated to hydrophobic leucine residues. The folded conformation of the DLeuβ-roll mutant is shown (PDB file: 5CVW) along with its peptide sequence with mutated positions in purple. (**b**) Exemplary pictures of hydrogel formed by MBP-DLeuβ-roll-MBP-DLeuβ-roll (**left**), and by the construct composed of DLeuβ-roll and PN406β-roll (**right**). The pictures on the right were taken under UV light to demonstrate the retention of the FITC-labeled target. Adapted with permission from Biomacromolecules, 2014, 15(10), pp 3617–3624. Copyright (2014) American Chemical Society and ACS Synth. Biol., 2017, 6(9), pp 1732–1741. Copyright (2017) American Chemical Society.

In a further follow-on study, the 8 positions on the opposing face of the β-roll were also mutated to leucine residues, resulting in the double-faced leucine mutant, DLeuβ-roll (Figure 4a) [46]. This rationally designed mutant had two hydrophobic surfaces available for cross-linking interactions. This mutant was fused to H and S domains, and it was shown that calcium-bound DLeuβ-roll was capable of forming hydrogels at a lower weight percentage (4 wt %) compared to the Leuβ-roll (6 wt %). Mean square displacement (MSD) plots at different calcium concentrations (0–10 mM) demonstrated that the mechanical properties of the H-S-DLeuβ-roll hydrogel could be tuned with calcium as well (Figure 3b).

### 3.2. DLeuβ-Roll as a Stand-Alone Crosslinking Domain

The utility of DLeuβ-roll was further explored by genetically constructing concatamers of DLeuβ-roll and maltose binding protein (MBP), eliminating the α-helical leucine zipper domain [46]. A clear transition in sample mechanical properties was observed upon calcium addition, indicating that DLeuβ-roll mutant was capable of establishing sufficient cross-linking associations by itself to initiate self-assembly in the presence of calcium. Thus, this mutant RTX domain was capable of serving as a stand-alone crosslinking domain.

The DLeuβ-roll was utilized in another hydrogel study, where this peptide was fused to an additional RTX domain mutant that was evolved to be capable of calcium-dependent target capture, the PN406β-roll [22]. The PN406β-roll (selected using an alternating positive and negative selection scheme (PN)) was evolved to bind to lysozyme only in its folded conformation, and this mutant was shown to reversibly capture and release the target protein upon calcium addition and removal [21]. A new polypeptide composed of DLeuβ-roll and PN406β-roll had a novel dual-functionality: calcium-dependent hydrogel formation, and calcium-dependent target protein binding and retention. In the presence of calcium, both hydrogel network assembly and fluorescein isothiocyanate (FITC)-labeled target capture were achieved, as shown in Figure 4b.

### 3.3. RTX Domain Fused to Elastomeric Proteins

RTX domain-based recombinant polypeptides were constructed by fusing the WTβ-roll peptide from CyA to GB1 domains and resilin-like sequences, whereby the former and latter provided superior solubility and crosslinking sites for network assembly, respectively [47]. In this study, hydrogel formation was achieved via a ruthenium-mediated photochemical crosslinking approach, independent of the presence of calcium. Following hydrogel construction, the biomaterial was subjected to 10 mM calcium and a decrease in hydrogel volume of 30% was observed. The conformational change of the β-roll upon calcium binding resulted in hydrogel compaction. Following ethylenediaminetetraacetic acid (EDTA) treatment, calcium ions were chelated and the samples swelled to their original volume. This process was reversible and full recovery was possible for at least three cycles. Thus, the constructed RTX-based hydrogel exhibited calcium responsive, tunable mechanical properties due to the transition of the RTX domain between alternate conformations, and the shrinking and swelling properties of the biomaterial were controlled by adjusting calcium concentrations. 

### 3.4. RTX Domain Fused to 6-Phospho-β-Galactosidase

In another study focusing on the design of different protein mesh networks, an RTX domain was fused between two 6-phospho-β-galactosidase (PGAL) molecules [48]. The RTX domain was used as a spacer and was composed of three β-roll helices with five repeats of the characteristic nonapeptide sequence taken from the serralysin protein of *S. Marcescens*. Electron microscopy was used to show that in the presence of calcium, the PGAL proteins were close to each other, as the RTX domain spacer remained short in its calcium bound state. Following the removal of calcium ions via EDTA, the proteins became separated by a range of distances from 3 nm to 13 nm, indicating that the RTX domain transitioned to its unfolded state, extending the space between the PGAL molecules. This study showed that RTX domains could be used to serve as calcium-responsive molecular springs, whereby the distances between network components can be controlled by calcium addition.

### 3.5. RTX Domain Conjugated to Palmitic Acid

A hydrogel responsive to both calcium and pH was constructed by single-tail peptide amphiphiles (PA) composed of a short RTX sequence and hydrophobic palmitic acid. These molecules were capable of self-assembly into nanofibers and subsequent gelation [49]. Three different RTX sequences were explored. The first one was based on the RTX sequence GGAGNDDLD. In the second and third versions, the aspartic acid residues were mutated to alanine resulting in PA2 (GGAGNADLD) and PA3 (GGAGNAALD). PA1 was selectively responsive to both pH and calcium, and formed rigid, viscoelastic hydrogels at a pH of 2 or with 2.5 mM calcium. Flip test and rheology data showed that PA2 was also capable of gelation in response to changes in pH and calcium addition, whereas PA3 could not form hydrogels at a pH of 2 and formed only weak networks upon addition of calcium. These results indicated that the aspartic acid residues were responsible for both pH sensitivity and calcium binding.

## 4. Summary and Conclusions 

There has been a good deal of interest in developing new protein-based biomaterials using native biomolecular interactions. RTX domains are useful for this approach, as they exhibit a well-defined and tunable conformational transition that is very specific to calcium. These domains have been used as controllable spacers between proteins, and as controllable domains in peptide amphiphile designs. We have shown that the mutagenesis of the exposed side chains on the faces of the β-roll domain can be used to design hydrophobic interfaces that assemble in a calcium-dependent fashion. These domains are contributing to the toolbox available for responsive biomaterial development and it is clear that the combination of these domains with other responsive components will lead to the development of new materials capabilities.
